# Ultrasound for Intra-Operative Detection of Peri-Centimetric Pulmonary Nodules in Uniportal Video-Assisted Thoracic Surgery (VATS): A Comparison with Conventional Techniques in Multiportal VATS

**DOI:** 10.3390/jcm13154448

**Published:** 2024-07-29

**Authors:** Sebastiano Angelo Bastone, Alexandro Patirelis, Matilde Luppichini, Vincenzo Ambrogi

**Affiliations:** 1 Department of Thoracic Surgery, Tor Vergata University Polyclinic, 00133 Rome, Italy; sebastianoangelo.bastone@ptvonline.it (S.A.B.); alexandro.patirelis@hotmail.it (A.P.); matilde.luppichini@ptvonline.it (M.L.); 2 Ph.D. Program in Applied Medical-Surgical Sciences, Department of Surgical Sciences, Tor Vergata University, 00133 Rome, Italy

**Keywords:** pulmonary nodule, pulmonary ultrasounds, intra-operative lung ultrasound, pulmonary nodule detection, uniportal VATS

## Abstract

**Background:** Video-assisted thoracic surgery (VATS) has become the gold-standard approach for lung resections. Given the impossibility of digital palpation, we witnessed the progressive development of peri-centimetric and deeply located pulmonary nodule alternative detection techniques. Intra-operative lung ultrasound is an increasingly effective diagnostic method, although only a few small studies have evaluated its accuracy. This study analyzed the effectiveness and sensitivity of uniportal VATS with intra-operative lung ultrasound (ILU), in comparison to multiportal VATS, for visualizing solitary and deep-sited pulmonary nodules. **Methods:** Patient data from October 2021 to October 2023, from a single center, were retrospectively gathered and analyzed. In total, 31 patients who received ILU-aided uniportal VATS (Group A) were matched for localization time, operative time, sensitivity, and post-operative complications, with 33 undergoing nodule detection with conventional techniques, such as manual or instrumental palpation, in multiportal VATS (Group B). Surgeries were carried out by the same team and ILU was performed by a certified operator. **Results:** Group A presented a significantly shorter time for nodule detection [median (IQR): 9 (8–10) vs. 14 (12.5–15) min; *p* < 0.001] and operative time [median (IQR): 33 (29–38) vs. 43 (39–47) min; *p* < 0.001]. All nodules were correctly localized and resected in Group A (sensitivity 100%), while three were missed in Group B (sensitivity 90.9%). Two patients in Group B presented with a prolonged air leak that was conservatively managed, compared to none in Group A, resulting in a post-operative morbidity rate of 6.1% vs. 0% (*p* = 0.16). **Conclusions:** ILU-aided uniportal VATS was faster and more effective than conventional techniques in multiportal VATS for nodule detection.

## 1. Introduction

According to the World Health Organization (WHO), lung cancer is the second most common cancer. It represents the first cause of death for tumor in men and the second cause of death in women every year [1].

The early identification of malignant nodules is mostly important for surgeons, and it is guaranteed in the pre-operative phase by the increasing resolution power of Computed Tomography (CT). Low dose CT has proved to be the most sensible exam to show lung nodules [2]. In addition, currently, more lung tumors are discovered at a very early stage thanks to the diffusion of lung cancer screening programs, thus increasing overall survival [3].

The recognition of possible lung cancers at a primordial stage allows for the performance of surgery with a curative intent with minimally invasive approaches, such as video-assisted thoracic surgery (VATS). The last evolution in this field is represented by uniportal VATS, which has shown to be non-inferior compared to multiportal VATS [4].

According to this attainment, the identification of small and deeply located nodules could become a major issue during uniportal VATS lung surgery, where the single small incision does not allow for the entry of the surgeon’s finger for digital exploration, making instrumental movements for the detection of the nodule more challenging.

In order to solve this limit, many techniques have been developed to guarantee a fast and accurate intra-operative localization of lung nodules, such as coils, lipiodol, nuclear isotopes, intra-operative injection of indocyanine green fluorescence, and pre-operative injection of drugs like methylene blue [5,6,7,8,9]. Nonetheless, these techniques are often complicated by procedural-related morbidity like pneumothorax, bleeding or air embolism, and elevated costs. Furthermore, these procedures usually have to be planned before surgery and the limited half-life of the used products could generate organizational problems [5,7,10]. Alternatively, these markers could be directly placed in hybrid operating rooms, which are not available in all hospitals [11].

Ultrasonography is routinely used for the study of subdiaphragmatic organs, and its application during abdominal surgery has been widely documented for decades [12]. On the contrary, its extensive use on lung parenchyma has often been discouraged due to the presence of soft tissue and the air interface causing image artifacts, which still represents a consistent obstacle to its effective popularization [13]. However, the possibility of applying this tool directly on a collapsed lung parenchyma after pulmonary selective intubation, and avoiding overlying the soft tissues thanks to the use of a completely endoscopic probe, has completely revolutionized its use.

Since the end of the previous century, some researchers have been successfully reporting the use of intra-operative lung ultrasonography (ILU) during thoracoscopy, taking advantage, as anticipated, of the lack of an air interface after pulmonary exclusion, in order to identify pulmonary nodules [14,15]. This technique further improved and started being adopted in minimally invasive approaches such as uniportal VATS, although with a limited number of patients [16].

This study aims to analyze the effectiveness of ILU in uniportal VATS in terms of operative times and sensitivity compared to the classic manual or instrumental palpation performed in multiportal VATS.

## 2. Materials and Methods

### 2.1. Patient Selection

This retrospective and monocentric study was based on the detection of pulmonary nodules during VATS lung resections. Patients were consecutively enrolled from October 2021 to October 2023 and they all underwent lung wedge resection for intra-operative histological examination either in uniportal VATS with ILU (group A) or in multiportal VATS (group B). All the procedures were performed by the same surgical team (operator and first assistant). The group allocation depended on the period of surgery—the procedures were initially performed in multiportal VATS from October 2021 to December 2022. Despite the long experience with uniportal VATS, we decided to perform these surgeries directly with multiple accesses after evaluating the small sizes and the deep location of the nodule in order to avoid excessive times and a too-forced traction on the lung to find the nodule. Thereafter, we started performing uniportal VATS for the resection of small and deeply located nodules soon after the arrival of an ultrasound machine equipped with an intra-operative probe in December 2022. All the ILU evaluations were performed by the same certified operator. Consequently, group B was made up of patients enrolled consecutively from October 2021 to December 2022, while group A was composed of patients operated on from December 2022 to October 2023. Patient data were retrieved from medical records and operative reports. They were stored in a dedicated database with the permission of our internal review board. Global and step-by-step operative times were available according to the program of data recording established in the operative room. Data collected were demographics (i.e., sex and age), features of the nodule (i.e., diameter, location, and final histology), type of surgery (i.e., uniportal or multiportal VATS lung resections), time to detection (time for identification of the nodule after pleural cavity entrance), operative time (from surgical incision to the end of wedge resection for intra-operative histological examination; we did not consider time for the completion of possible lobectomy after frozen section), and need of additional wedge resection for missed nodule and post-operative complications developed within 30 days after surgery. The inclusion criteria were age over 18 years, the presence of a suspicious less than 2 cm completely solid lung nodule, a distance between the nodule and the pleural surface of at least 2 cm, and a pulmonary wedge resection for intra-operative histological examination performed in VATS. Exclusion criteria were conversion to thoracotomy, pulmonary nodules greater than or equal to 2 cm, part-solid nodules, and a distance between visceral pleura and nodule < 2 cm. Patients with a high grade of pulmonary emphysema and a significant air-trapping, hindering pulmonary collapse despite bronchial selective intubation, were also excluded from the study.

### 2.2. Surgical Details

**Figure 1:**
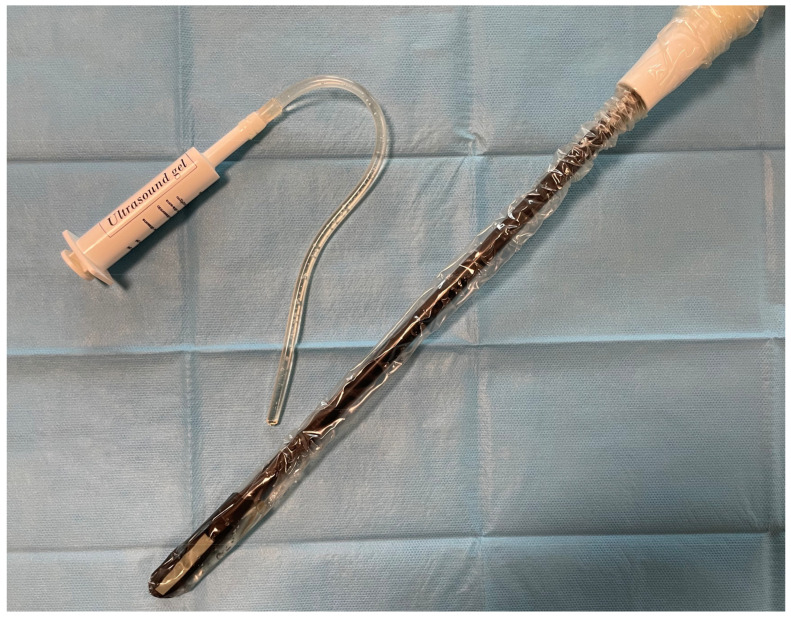
Intra-operative probe covered with a sterile plastic bag with gel to improve echoic resolution.

The ultrasound probe is about 43 cm long and it is equipped with a distal 10 cm articulated head that allows 90° up–down and left–right movements, providing an easy exploration of the whole lung, despite the single port approach. Piezoelectric crystals are distributed in the last 4 cm of the probe only on one face, and the frequency of the probe had a 13–4 MHz range. In our experience, the best setting for lung nodule detection is an abdominal preset with gain at 70% and a depth between 50 mm and 60 mm. We pre-operatively put a sterile cover on the probe, although the instrument could be self-sterilizable (Figure 1). In this way, it is possible to fill the protective plastic bag with gel, thus improving the echoic resolution.

All procedures were performed with the patient in lateral decubitus with the side affected by the pathology facing upwards. For all patients, the *latissimus dorsi* muscle was identified and spared. The serratus muscle was always identified and incised along its muscle bundles until reaching the costal plane. After having evidenced the chest wall and choosing the intercostal space, we entered the pleural space for an approximate length of 3 cm through the inferior edge of the rib. An Alexis-type atraumatic soft tissue retractor was therefore positioned.

**Figure 2:**
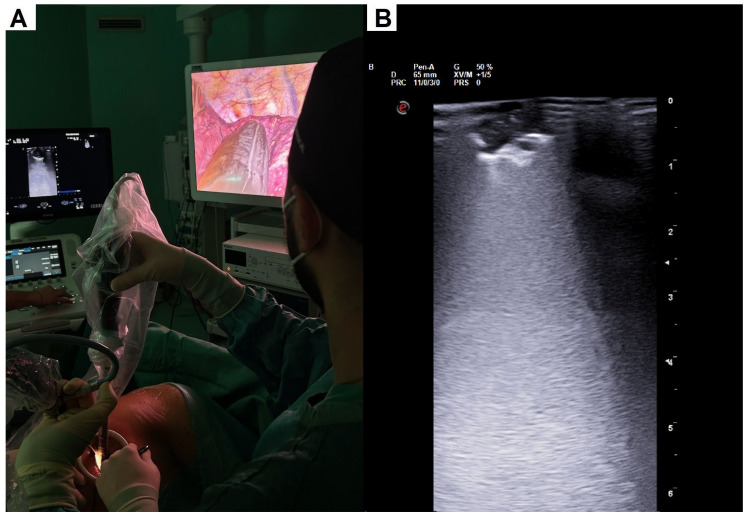
(**A**) Nodule identification with intra-operative lung ultrasound in uniportal VATS. Instrumental disposition from top to bottom—camera, ILU probe, and retraction forceps. (**B**) Ultrasound visualization of a lung nodule, with irregularity of the pleural line and posterior hyperechoic reinforcement.

Identification of the pulmonary nodule was carried out through manual palpation or with a mounted swab in patients undergoing multiportal VATS. In the case of patients undergoing uniportal VATS, the nodule was localized using the intra-operative ultrasound probe. The camera was placed in the highest part of the incision, while the probe was placed in the middle part, and the retraction forceps were placed in the lowest part (Figure 2A). This arrangement was associated with a light pressure on the lung and latero-lateral rotatory movements. These movements allowed us to easily explore the entire lung parenchyma. The presence of the nodule was ascertained at ultrasound display by evidencing the typical signs of the pulmonary nodule such as interruption or irregularity of the pleural line associated with posterior hyperechoic reinforcement (Figure 2B). Once the nodule was localized and following a double check with a mounted swab, we then performed wedge resection.

The lung resection was completed by placing mechanized linear staplers in sequence and the specimen was sent for intra-operative histological examination. In the case of no parenchymal alterations being found by the pathologist in the first specimen, an additional wedge resection was performed in order to obtain an intra-operative diagnosis. If the histological report was consistent for non-small-cell lung cancer, the patient was submitted to lobectomy or segmentectomy according to pre-operative pulmonary function. Conversely, in the case of pulmonary metastasis or benign lesion, no more extensive resections were performed. At the end of the surgery, a single chest drainage was placed in all patients.

### 2.3. Statistical Analysis

Statistical analysis was performed with SPSS (IBM Corp. Released 2016. IBM SPSS Statistics, Version 26.0; Armonk, NY, USA: IBM Corp.). A *p*-value less than 0.050 was considered as statistically significant. Categorical variables were reported as frequencies with percentage. Continuous ones were presented as median with interquartile range (IQR) due to a not-normal distribution. Descriptive analysis of data was executed using Pearson’s χ-square for categorical variables. For continuous variables, the Mann–Whitney non-parametric test was preferred due to the not-normal distribution.

## 3. Results

### 3.1. Demographics and Clinical Characteristics

Out of a total of 64 patients enrolled on the study, 31 (Group A) underwent wedge resection in uniportal VATS with ILU for nodule identification. The remaining 33 (Group B) patients received a wedge resection in multiportal VATS with traditional manual or instrumental palpation for the detection of the suspected lesion.

The main demographic and clinical features of the population are reported in Table 1. The groups were homogeneous for gender distribution, median age of 70 (IQR 66–76) vs. 71 (IQR 65.7–75.2) years, median nodule diameter of 11 (IQR 9–13) vs. 11 (IQR 9–12) mm, and upper lobe location (54.83% vs. 57.57%). No significant differences resulted in histology distribution, which was prevalently malignant (80.7% vs. 72.7%).

### 3.2. Intra-Operative Results and Post-Operative Complications

The results about operative times and intra- and post-operative outcomes are summarized in Table 2. The detection time was significantly shorter in Group A, with a median time of 9 (IQR 8–10) min versus 14 (IQR 12.5–15) min (*p* < 0.001). Similarly, the global operative time was significantly shorter in Group A with a median time of 33 (IQR 29–38) min vs. 43 (IQR 39–47) min (*p* < 0.001). No significant difference was found between the groups with reference to the distance of the nodule from the pleural surface (*p* = 0.54).

In Group A, ILU allowed for the detection of all nodules at the first attempt, with a sensitivity of 100.0%. Conversely, in Group B, three patients (9.1%) required an additional wedge resection, since no nodule had been found in the first specimen at intra-operative histological examination, with a final sensitivity of 90.1%. However, the comparison of the two groups for this parameter was not statistically significant, at *p* = 0.086. We did not report the sonographic characteristics of the nodule on the operative notes, as the main aim was only the detection of the lesion.

When considering post-operative outcomes, no cases of post-operative morbidity were reported in Group A. Conversely, we recorded two cases of prolonged (more than 10 days) air leaks in Group B, with a final post-operative morbidity of 6.1%. Both cases were managed conservatively, and patients were discharged with a Heimlich valve. Nevertheless, no significant difference was found (*p* = 0.16).

## 4. Discussion

The identification of peri-centimetric, especially if deeply located, pulmonary nodules has been an increasingly prevalent issue in recent years. After the diffusion of minimally invasive techniques, such as VATS, the impossibility for surgeons to properly use their hands for the individuation of the small lesion makes the problem even more sensitive.

Lung ultrasound is an increasingly effective and cheaper diagnostic method that can be performed in real time directly during surgery.

Our study demonstrates how this technique could be easily associable with a uniportal thoracoscopic approach, achieving an excellent detection rate and shorter operative times not only explainable by the single surgical access instead of multiple ones. Our data about uniportal VATS with ILU also showed no post-operative complications, such as prolonged air leaks; this was as a result, according to our opinion, of less manipulation of the parenchyma with rigid instrumentation and thanks to the probe with an articulated head (up–down and lateral movements) allowing us to reach close to all of the parenchyma, avoiding an excessive traction. Although associated with less invasiveness, our data suggested a trend in ultrasound to have a higher sensitivity for the detention of the nodule, considering that we did not experience the necessity to repeat a wedge resection for missing nodules in any case of the uniportal group. The lack of statistical significance could probably be due to the limited sample.

In the literature, ILU performed during VATS was demonstrated to be a reproducible and assured method for visualization and localization of small and deeply located pulmonary nodules that had not previously been found on digital palpation [17,18], reducing the risk of conversion to thoracotomy or avoiding an upfront “blind” lobectomy without histological diagnosis [19].

Unlike other methods applied for the localization of peri-centimetric and deep-sited lung nodules such as coils, lipiodol, nuclear isotopes, or the pre-operative injection of drugs like methylene blue [5,6,7,8,9,10], ILU is cheap, fast to perform, and does not require the involvement of another professional figure beyond the surgeon. In addition, as our study demonstrated, it is a safe procedure and it is not related to an increased risk of complications like bleeding or air embolism, which is different from other methods [5,7].

The feasibility of ILU associated with uniportal VATS for the detection of peri-centimetric and peripheral lung nodules has already been demonstrated by Rocco et al. [16]. Nevertheless, this study was conducted only on two patients with peripheral nodules. To the best of our knowledge, our study was the first one involving a consistent sample size of patients submitted to uniportal VATS and comparing the technique with traditional manual and instrumental palpation in multiportal VATS.

ILU also allows surgeons not only to detect the nodule, but also to perform a first evaluation of some parameters such as echogenicity, margins, presence of calcifications, and more precise nodule sizes [20], thus helping in predicting the risk of malignancy and evaluating the extension of surgical resection. Indeed, the presence of a heterogeneous echogenic pattern and of ill-defined margins increase the probabilities that the nodule could be malignant [10]. Moreover, an additional tool that can be used for the intra-operative characterization of suspected pulmonary nodules, as recently suggested by Schauer et al., is contrast-enhanced ultrasound (CEUS) with microbubbles. This technique, already applied in hepatic surgery, allows for the evaluation of nodule microvascularization, and has been revealed to be suited to make a first differential diagnosis among primary lung cancers, pulmonary metastases, and benign lesions [21]. Beyond the possibility of obtaining an initial orientation about the nature of the lesion, the use of ILU could help in achieving samples with clear margins and higher diagnostic rates [22]. In particular, it was revealed to be feasible and effective in the detection of ground glass opacities, which are usually difficult to identify, as well as giving tactile feedback in the definition of their margins [23].

This study is limited by the relatively small number of patients and by its retrospective nature. A real randomized prospective study is highly desirable and we plan to set it in motion shortly.

As far as concerns the technique by itself, we can say that ILU has certain limitations. This procedure still has a very subjective interpretation, providing different results according to the different operators. In order to gain reproducible and comparable results among different centers, ILU requires a very skilled and expert operator, which is, at the moment, not readily available for every operative team. Furthermore, given the significant air-trapping despite pulmonary selective exclusion, ILU can provide unsatisfactory and non-evaluable findings in severely emphysematous patients, which represent a consistent share of patients undergoing surgery for lung nodules.

However, after these promising results, we hope to enlarge our experience associating ILU with robot-assisted thoracic surgery (RATS). For this purpose, we hypothesized the development of an appositely dedicated robotic ultrasound probe. This could hopefully mitigate the limitation of tactile feedback during RATS operations.

## 5. Conclusions

In conclusion, we can affirm that ILU is a very important and effective tool for the intra-operative nodule detection of localized peri-centimetric and deeply located nodules, especially in minimally invasive techniques such as uniportal VATS. We demonstrated faster operation times and identification of the nodules and a trend of better sensitivity compared to conventional nodule identification techniques in multiportal VATS.

## Figures and Tables

**Table 1:** Demographic and clinical characteristics of the enrolled population. IQR: interquartile range.

Variable	Group A (*n* = 31)	Group B (*n* = 33)	*p*-Value
Median age, years (IQR)	70 (66–76)	71 (65.7–75.2)	0.99
Gender, *n* (%)			0.81
male	16 (51.6%)	18 (54.5%)	
female	15 (48.4%)	15 (45.5%)	
Nodule diameter, mm (IQR)	11 (9–13)	11 (9–12)	0.92
Nodule location, *n* (%)			0.85
right upper lobe	8 (25.8%)	9 (27.3%)	
middle lobe	2 (6.5%)	3 (9.1%)	
right inferior lobe	4 (12.9%)	6 (18.2%)	
left upper lobe	9 (29.0%)	10 (30.3%)	
left inferior lobe	8 (25.8%)	5 (15.1%)	
Final histology, *n* (%)			0.47
Adenocarcinoma	14 (45.2%)	18 (54.5%)	
Squamous cell carcinoma	9 (29.0%)	5 (15.2%)	
Pulmonary metastasis	2 (6.5%)	1 (3.0%)	
Benign lesion	6 (19.3%)	9 (27.3%)	

**Table 2:** Operative times and intra- and post-operative outcomes. IQR: interquartile range.

Variable	Group A (*n* = 31)	Group B (*n* = 33)	*p*-Value
Detection time, minutes (IQR)	9 (8–10)	14 (12.5–15)	<0.001
Operative time, minutes (IQR)	33 (29–38)	43 (39–47)	<0.001
Nodule distance from pleural surface, mm (IQR)	27 (25–28)	26 (24–28)	0.54
Need of additional wedge, *n* (%)			0.086
yes	0 (0.0%)	3 (9.1%)	
no	31 (100.0%)	30 (90.9%)	
Post-operative complication, *n* (%)			0.16
yes	0 (0.0%)	2 (6.1%)	
no	31 (100.0%)	31 (93.9%)	

## Data Availability

Data are contained within the article.

## References

[1] Sung H., Ferlay J., Siegel R.L., Laversanne M., Soerjomataram I., Jemal A., Bray F. (2021). Global Cancer Statistics 2020: GLOBOCAN Estimates of Incidence and Mortality Worldwide for 36 Cancers in 185 Countries. CA Cancer J. Clin..

[2] Adams S.J., Stone E., Baldwin D.R., Vliegenthart R., Lee P., Fintelmann F.J. (2023). Lung cancer screening. Lancet.

[3] Aberle D.R., Adams A.M., Berg C.D., Black W.C., Clapp J.D., Fagerstrom R.M., Gareen I.F., Gatsonis C., Marcus P.M., National Lung Screening Trial Research Team (2011). Reduced lung-cancer mortality with low-dose computed tomographic screening. N. Engl. J. Med..

[4] Zhao R., Shi Z., Cheng S. (2019). Uniport video assisted thoracoscopic surgery (U-VATS) exhibits increased feasibility, non-inferior tolerance, and equal efficiency compared with multiport VATS and open thoracotomy in the elderly non-small cell lung cancer patients at early stage. Medicine.

[5] Sui X., Zhao H., Yang F., Li J.L., Wang J. (2015). Computed tomography guided microcoil localization for pulmonary small nodules and ground-glass opacity prior to thoracoscopic resection. J. Thorac. Dis..

[6] Mogi A., Yajima T., Tomizawa K., Onozato R., Tanaka S., Kuwano H. (2015). Video-Assisted Thoracoscopic Surgery after Preoperative CT-Guided Lipiodol Marking of Small or Impalpable Pulmonary Nodules. Ann. Thorac. Cardiovasc. Surg..

[7] Bellomi M., Veronesi G., Trifirò G., Brambilla S., Bonello L., Preda L., Casiraghi M., Borri A., Paganelli G., Spaggiari L. (2010). Computed tomography-guided preoperative radiotracer localization of nonpalpable lung nodules. Ann. Thorac. Surg..

[8] Nagai K., Kuriyama K., Inoue A., Yoshida Y., Takami K. (2018). Computed tomography-guided preoperative localization of small lung nodules with indocyanine green. Acta Radiol..

[9] Findik G., Demiröz S.M., Apaydın S.M.K., Ertürk H., Biri S., Incekara F., Aydogdu K., Kaya S. (2017). Computed Tomography-Guided Methylene Blue Labeling Prior to Thoracoscopic Resection of Small Deeply Placed Pulmonary Nodules. Do We Really Need Palpation?. Thorac. Cardiovasc. Surg..

[10] Matsumoto S., Hirata T., Ogawa E., Fukuse T., Ueda H., Koyama T., Nakamura T., Wada H. (2004). Ultrasonographic evaluation of small nodules in the peripheral lung during video-assisted thoracic surgery (VATS). Eur. J. Cardiothorac. Surg..

[11] Partlow J., Thomas S., Nicolini M., Greeno S., Schroeder C. (2024). Image-Guided VATS in the Hybrid Operation Room Facilitates Early Diagnosis and Concurrent Treatment of Subcentimeter Nonpalpable Lung Nodules. Innovations.

[12] Machi J., Sigel B., Zaren H.A., Schwartz J., Hosokawa T., Kitamura H., Kolecki R.V. (1993). Technique of ultrasound examination during laparoscopic cholecystectomy. Surg. Endosc..

[13] Sperandeo M., Rotondo A., Guglielmi G., Catalano D., Feragalli B., Trovato G.M. (2014). Transthoracic ultrasound in the assessment of pleural and pulmonary diseases: Use and limitations. Radiol. Med..

[14] Greenfield A.L., Steiner R.M., Liu J.B., Cohn H.E., Goldberg B.B., Rawool N.M., Merton D.A. (1997). Sonographic guidance for the localization of peripheral pulmonary nodules during thoracoscopy. Am. J. Roentgenol..

[15] Santambrogio R., Montorsi M., Bianchi P., Mantovani A., Ghelma F., Mezzetti M. (1999). Intraoperative ultrasound during thoracoscopic procedures for solitary pulmonary nodules. Ann. Thorac. Surg..

[16] Rocco G., Cicalese M., La Manna C., La Rocca A., Martucci N., Salvi R. (2011). Ultrasonographic identification of peripheral pulmonary nodules through uniportal video-assisted thoracic surgery. Ann. Thorac. Surg..

[17] Taurchini M., Quarato C.M.I., Frongillo E.M., Ferretti G.M., Cipriani C., Bizzarri M., Foschino Barbaro M.P., Lacedonia D., Simeone A., Graziano P. (2021). Intraoperative Lung Ultrasound (ILU) for the Assessment of Pulmonary Nodules. Diagnostics.

[18] Huang Y.H., Chen K.C., Chen J.S. (2019). Ultrasound for intraoperative localization of lung nodules during thoracoscopic surgery. Ann. Transl. Med..

[19] Khereba M., Ferraro P., Duranceau A., Martin J., Goudie E., Thiffault V., Liberman M. (2012). Thoracoscopic localization of intraparenchymal pulmonary nodules using direct intracavitary thoracoscopic ultrasonography prevents conversion of VATS procedures to thoracotomy in selected patients. J. Thorac. Cardiovasc. Surg..

[20] Gambardella C., Messina G., Pica D.G., Bove M., Capasso F., Mirra R., Natale G., D’Alba F.P., Caputo A., Leonardi B. (2023). Intraoperative lung ultrasound improves subcentimetric pulmonary nodule localization during VATS: Results of a retrospective analysis. Thorac. Cancer..

[21] Schauer M.I., Jung E.M., Platz Batista da Silva N., Akers M., Loch E., Markowiak T., Piler T., Larisch C., Neu R., Stroszczynski C. (2023). Intraoperative Contrast-Enhanced Ultrasonography (Io-CEUS) in Minimally Invasive Thoracic Surgery for Characterization of Pulmonary Tumours: A Clinical Feasibility Study. Cancers.

[22] Sperandeo M., Venuti M., Quarato C.M.I. (2020). Uniportal versus multiportal video-assisted thoracic surgery for lung cancer: Safety and advantages in employing complementary intraoperative lung ultrasound. J. Thorac. Dis..

[23] Messina G., Bove M., Natale G., Noro A., Martone M., Opromolla G., Di Filippo V., Leonardi B., Fasano M., Polito R. (2022). Ultrasound location of ground-glass opacity during thoracoscopic surgery. Interact. Cardiovasc. Thorac. Surg..

